# The Moderating Effect of Social Support on the Association Between Healthcare Discrimination and Quality of Life in Persons with Type 2 Diabetes †

**DOI:** 10.3390/healthcare14010031

**Published:** 2025-12-22

**Authors:** Emmanuel Ekpor, Emefa Awo Adawudu, Samuel Adu Agyen, Debby Syahru Romadlon, Samuel Akyirem

**Affiliations:** 1 School of Psychology, Institute for Health Transformation, Deakin University, Geelong, VIC 3220, Australia; 2 The Australian Centre for Behavioural Research in Diabetes, Diabetes Victoria, Carlton, VIC 3053, Australia; 3 Elaine Marieb College of Nursing, University of Massachusetts, Amherst, MA 01003, USA; eadawudu@umass.edu; 4 Korle Bu Teaching Hospital, Accra P.O. Box 77, Ghana; 5 Faculty of Nursing, Chulalongkorn University, Bangkok 10330, Thailand; 6 Yale School of Nursing, Yale University, New Haven, CT 06477, USA

**Keywords:** healthcare discrimination, social support, type 2 diabetes, quality of life

## Abstract

**Background/Objectives:** Healthcare discrimination poses significant challenges to the health-related quality of life (HRQoL) of people living with type 2 diabetes (T2D). However, the role of social support in alleviating these effects has not been fully explored. Drawing on Cohen and Wills’ social support buffering model, this study examined whether social support moderates the association between healthcare discrimination and HRQoL among individuals with T2D. **Methods:** We analyzed data from 5180 adults with T2D enrolled in the *All of Us* Research Program. Healthcare discrimination was assessed using the modified Everyday Discrimination Scale (mEDS), social support with the Medical Outcomes Study Social Support Survey (MOS-SSS), and HRQoL (physical and mental domains) with the PROMIS Global Health Scale. Moderation analyses were conducted through linear regression models. **Results:** Greater exposure to healthcare discrimination was associated with poorer physical and mental HRQoL. Social support demonstrated a significant moderating effect on mental HRQoL: as social support increased, the negative association between healthcare discrimination and mental well-being weakened. However, this buffering effect was not observed for physical HRQoL. **Conclusions:** Findings suggest that social support can mitigate the adverse mental health consequences of healthcare discrimination among individuals with T2D. Interventions aimed at strengthening social support networks warrant investigation as potential strategies to improve the mental HRQoL of people with T2D who encounter discrimination in healthcare environments.

## 1. Introduction

Discrimination in healthcare remains a pervasive issue that compromises individuals’ well-being [1]. In the United States (US) alone, approximately 21% of adults report at least one encounter of discriminatory treatment in healthcare settings [2], ranging from dismissive communication to unequal treatment or restricted access to necessary care [3,4]. Such experiences not only strain therapeutic relationships but also compromise health outcomes. For instance, discrimination in healthcare has been linked to higher levels of depression, poorer self-rated health [5], increased cardiometabolic risk [6], and a greater likelihood of new or worsened disability over a period of four years [7].

Discrimination in healthcare is a significant issue among people with type 2 diabetes (T2D). Recent international consensus reports that 1 in 5 adults with T2D experience discrimination, including bias and negative behaviors from healthcare professionals [8]. Research shows that healthcare professionals sometimes hold “unrealistic” expectations, often attributing suboptimal health outcomes solely to individual behavior, while overlooking systemic factors that contribute to T2D management challenges [9]. Others may dismiss the health concerns of persons with T2D due to negative assumptions about their health behaviors [10,11]. These discriminatory attitudes can serve as barriers to effective diabetes care, as individuals may avoid discussing their condition openly or even skip essential healthcare appointments.

Although healthcare discrimination is known to adversely affect health outcomes [5,6,7], its impact in the diabetes context remains insufficiently understood. Existing studies have largely focused on the experience and frequency of discriminatory encounters [9,10,11]. Among the limited studies exploring consequences, healthcare discrimination has been shown to weaken therapeutic relationships, which in turn was associated with lower diabetes care quality ratings [12]. Healthcare discrimination has also been associated with poorer physical function, suboptimal glycemic levels, and a higher incidence of diabetes-related complications [13,14]. Although these outcomes are significant indicators of health, there is a need for further investigation into how discrimination in healthcare affects health-related quality of life (HRQoL) in people with diabetes, and the potential factors that may alleviate these harmful effects. Furthermore, key sociodemographic and health-related factors (e.g., age, sex, race or ethnicity, education, income, and body mass index [BMI]) are known predictors of HRQoL in people with T2D [15]. Accounting for these factors is therefore essential to clarify the unique association between discrimination and HRQoL.

Social support, defined as the perception of having a reliable network to depend on [16], can moderate the effects of discrimination on health outcomes. According to Cohen and Wills (1985), the buffering model holds that social support can mitigate the negative impact of stressors such as discrimination [17]. Prior studies have confirmed this moderating role on the adverse effect of discrimination on psychological distress among African American adults [18] and on the general well-being among Korean adults [16]. However, the potential moderating effect of social support on the relationship between healthcare discrimination and HRQoL in persons with T2D has not been examined to the best of our knowledge.

**Figure 1:**
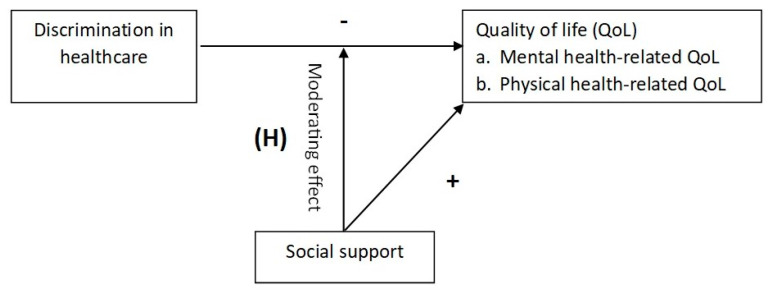
Hypothesized (H) pathway of the moderating effect of social support on the relationship between healthcare discrimination and mental and physical HRQoL.

Informed by Cohen and Wills’ social support buffering model, we aimed to examine whether social support moderates the association between healthcare discrimination and HRQoL among individuals with T2D. We hypothesized that (Figure 1), among people with T2D: (a) increased discrimination in healthcare will be negatively associated with mental and physical HRQoL, (b) higher social support will be positively associated with mental and physical HRQoL, and (c) social support will significantly buffer against the adverse impact of discrimination in healthcare on mental and physical HRQoL.

## 2. Materials and Methods

### 2.1. Study Design

We conducted a cross-sectional analysis on survey data obtained from the *All of Us* (*AoU*) research program (https://allofus.nih.gov/), a large-scale precision health initiative led by the National Institutes of Health (NIH). The goal of this research program is to recruit at least 1 million adults in the US, with recruitment strategies mostly targeting individuals from medically underrepresented backgrounds, including racial and ethnic, and sexual and gender minorities [19]. At enrollment, participants complete a core sociodemographic questionnaire and are subsequently invited to respond to additional surveys covering areas such as personal and family health history, overall health status, COVID-19 experiences, lifestyle behaviors, and social determinants of health (SDH). Participants may also opt to visit designated clinical sites for physical assessment, specimen collection, and genomic testing. They have the opportunity to share their Fitbit and electronic health records data with the research program [19]. The institutional review board of the *All of Us* Research Program approved the study protocol for this research program. In the present study, we used the basic, overall health, and SDH surveys, as well as on-site height and weight measurement data.

### 2.2. Selection of Study Cohort

Participants for this study were selected using the *All of Us* Researcher Workbench, a cloud-based interactive platform [19]. We used the Controlled Tier Curated Data Repository v7. Participants were included in the present analysis if they self-reported having T2D, had completed the SDH survey (which contained items for measuring discrimination in medical settings), were 18 years or older, and had complete data on all variables of interest. Overall, 17,507 participants self-reported that they had T2D. Out of this, 9964 completed the SDH survey. After excluding participants with missing data on variables of interest, 5180 participants remained (representing 51.99% of the SDH survey completers), which formed the final analytic sample.

### 2.3. Predictor Variable

The predictor variable for the current analyses was healthcare discrimination measured by the modified Everyday Discrimination Scale (mEDS). The mEDS was administered as part of the SDH survey of the *AoU* program [20]. The mEDS measures the experience of discrimination in the medical settings using 7 items that are rated on a 5-point scale (1—Never, 2—Rarely, 3—Sometimes, 4—Most of the time, 5—Always). Some representative items of the mEDS include “How often do you receive poorer service than others when you go to a doctor’s office or other healthcare provider?” and “How often do you feel like a doctor or nurse is not listening to what you were saying when you go to a doctor’s office or other healthcare provider?”. The total discrimination score was calculated by summing individual item scores for each participant, with higher scores signifying greater experience of discrimination in medical settings. The Cronbach’s alpha for the mEDS in this study was 0.90.

### 2.4. Outcome Variables

The current analyses included two outcome variables: mental HRQoL and physical HRQoL. These two outcomes were measured by the two subscales of the Patient Reported Outcomes Measurement Information System (PROMIS) Global Health scale [21]. The PROMIS was administered as part of the “Overall Health” survey of the *AoU* program. Each of the outcomes was measured with four items rated on a 5-point Likert scale [22]. Some representative questions on the mental and physical HRQoL subscales include: “In general, how would you rate your mental health, including your mood and your ability to think?” and “In the past 7 days, how would you rate your pain on average?”, respectively. The total score was calculated by summing the items for each subscale. Higher scores reflect better mental and physical HRQoL. The Cronbach’s alpha for the mental and physical subscales in this study was 0.85 and 0.76, respectively.

### 2.5. Moderator Variable

The moderator variable for this study was social support, measured using the Medical Outcomes Study Social Support Survey (MOS-SSS) [23]. The MOS-SSS was administered to *AoU* research participants as part of the SDH survey. The original MOS-SSS has 19 items. However, in the *AoU* program, the brief 8-item version was administered. The items of the scale are rated on a 5-point Likert scale (1—“None of the time”, 2—“A little of the time”, 3—“Some of the time”, 4—“Most of the time”, 5—“All of the time”). The 8-item brief MOS-SSS has been shown to have good psychometric properties, comparable to the full scale [24]. Some items on the brief MOS-SSS include “How often do you have someone to help you if you were confined to bed?” and “How often do you have someone to have a good time with?”. The total score is calculated by averaging all scores (range: 1–5). As recommended by the original developers of the MOS-SSS, the total scores were transformed to a 0–100 scale by the formula:

$$\frac{mean\;score-1}{4}\;\times \;100$$

Higher scores for the MOS-SSS signify greater perceived social support. The Cronbach’s alpha in this study was 0.95.

### 2.6. Covariates

The following covariates were included in this study: age, BMI, sex assigned at birth (i.e., male and female), race or ethnicity (categorized as Non-Hispanic White, Non-Hispanic Black, Hispanic, Asian, Others), marital status (categorized as married or living with a partner, single, and widowed/separated/divorced), level of education (categorized as high school or less, some college education, college degree, and advanced degree), and annual income (categorized as less than $35,000, $35,000–$74,999, $75,000–99,999, and $100,000 or more). These covariates were included because of their known association with HRQoL and discrimination [15,25], allowing us to account for potential confounders in our regression models.

### 2.7. Data Analysis

Data were analyzed with the R programming language (https://www.r-project.org/). We first summarized data by computing descriptive statistics such as means, standard deviations, proportions, and frequencies. Second, we assessed the association between healthcare discrimination and mental and physical HRQoL using multivariable linear regression. Two separate regression models (models 1a and 2a) were developed, one for each outcome measure (i.e., physical and mental HRQoL) while adjusting for all covariates. Third, to test the moderation effect of social support, we added the social support variable as an interaction term with the discrimination variable to each of the linear regression models (models 1b and 2b). Lastly, we performed slope analysis using Hayes’ PROCESS model 1 [26] and generated a Johnson-Neyman plot to help visualize and interpret the moderation effect of social support. Missing data were handled using complete case analysis, in which participants with missing values for any variables required in the analysis were excluded. No imputation was performed. A 2-sided *p*-value of ≤5% was considered statistically significant.

## 3. Results

### 3.1. Sample Characteristics

Table 1 shows the characteristics of participants included in this study. Participants had an average age of 61.69 years (SD = 11.27). The majority of participants were female (n = 2814, 52.32%) and identified as Non-Hispanic White (n = 3804, 73.44%). Most participants were either married or living with a partner (57.92%), had a household income below $35,000 (n = 1579, 30.48%), and had attained some level of college education (n = 1697, 32.76%). The average BMI of the sample was 34.36 (SD = 7.69) kg/m^2^.

### 3.2. Findings from the Multivariable Linear Regression

**Figure 2:**
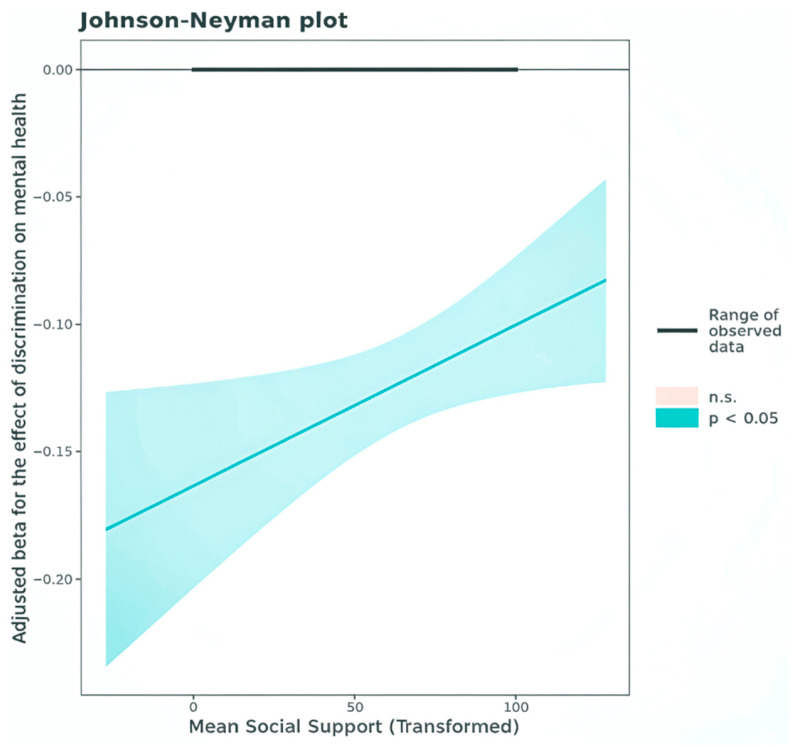
The moderating effect of social support on the association between healthcare discrimination and mental HRQoL.

Higher levels of healthcare discrimination were significantly associated with poorer physical HRQoL (adjusted beta = −0.10; 95% CI: −0.11, −0.08) and mental HRQoL (adjusted beta = −0.12; 95% CI: −0.14, −0.10). Greater social support was independently related to higher physical and mental HRQoL scores. Social support also demonstrated a significant moderating effect on mental HRQoL: as social support increased, the negative association between healthcare discrimination and mental HRQoL weakened (Figure 2, Table 2 and Table 3). In contrast, no significant moderation effect was observed for social support in the association between healthcare discrimination and the physical HRQoL domain. Beyond the focal variables, higher income and educational attainment, as well as older age, were associated with better mental and physical HRQoL, whereas higher BMI was linked to poorer outcomes across both domains (Table 2 and Table 3). It should be noted that the relatively high *R*^2^ values observed in Table 2 and Table 3 are due to the several significant covariates included in the models.

The result of the simple slope analysis (Table 4) reveals that while higher levels of discrimination are associated with lower mental HRQoL across low, average, and high levels of social support, the strength of the association decreases slightly as social support increases. Additionally, according to the Johson-Neyman plot shown in Figure 2, social support is a significant moderator of the association between discrimination and mental HRQoL only when social support values are less than 165.12.

## 4. Discussion

Guided by Cohen and Wills’ buffering model, this study used regression-based moderation analyses to examine the direct and interactive effects of healthcare discrimination and social support on mental and physical HRQoL among individuals with T2D. We tested the hypothesis that (a) increased discrimination in healthcare will be negatively associated with mental and physical HRQoL, (b) higher social support will be positively associated with mental and physical HRQoL, and (c) social support will significantly buffer against the adverse impact of discrimination in healthcare on mental and physical HRQoL.

Our first two hypotheses were confirmed. Although the regression coefficients were modest, they indicated significant direct effects, with higher discrimination predicting lower mental and physical HRQoL, and higher social support predicting better mental and physical HRQoL. This is consistent with prior research [27,28,29,30]. In the U.S., for instance, perceived discrimination was closely associated with stressful life events, which in turn, adversely affected mental health outcomes for individuals with T2D [27]. Likewise, the internalization of discriminatory treatment among individuals with T2D is found to be associated with physical HRQoL determinants such as higher HbA1c levels and lower levels of self-management [28]. Additionally, consistent with prior evidence, individuals with T2D who have strong social support tend to experience overall better HRQoL and clinical outcomes [29,30], underscoring the essential role of supportive interpersonal relationships in promoting well-being.

Our third hypothesis was partially supported. The interaction term between social support and healthcare discrimination was statistically significant for mental HRQoL, indicating a moderation effect. In contrast, the interaction term was non-significant for physical HRQoL, suggesting that social support may not necessarily buffer the impact of discrimination on physical health. While a recent study has reported a general moderating effect of social support on discrimination (differential treatment) in people with T2D, impacts on mental and physical HRQoL were not analyzed separately [31]. Thus, our study provides a nuanced understanding of the role of social support across different dimensions of health.

Several mechanisms may explain why the moderation effect emerged only for mental HRQoL. For example, social support—such as emotional reassurance, shared understanding, and empathy—can serve as a buffer against psychological stress, helping individuals process negative experiences and reducing the mental health burden of discrimination [18]. However, these supportive interactions may not directly alleviate physical symptoms or improve physical HRQoL. Also, social support often aids in cognitive appraisal, which is crucial for mental health [32]. Through supportive relationships, individuals may adopt alternative perspectives, helping them interpret discriminatory events less negatively, thereby reducing their psychological impact [33]. This cognitive reframing may enhance mental HRQoL but does not necessarily translate into physical health changes, which often require medical interventions beyond cognitive coping. Discrimination can impact mental and physical health through different pathways. For example, research has shown that mental health outcomes are closely tied to the emotional strain from discrimination in people with T2D [27]. Thus, while social support may alleviate the emotional toll on mental health, it might not sufficiently address these indirect, behavior-driven pathways that affect physical health.

## 5. Strengths and Limitations

This study’s strengths include the large sample size, which bolsters the statistical power of the findings. Additionally, this study tested a theory-driven hypothesis of the moderating effect of social support, providing valuable insights into its potential protective effect against discrimination among people with T2D, which has practical implications for healthcare interventions. Despite these strengths, several limitations exist. First, the study’s cross-sectional design limits causal inference, making it unclear whether discrimination, social support, or HRQoL changes over time. Second, the use of self-reported measures may introduce recall and social desirability bias. Third, although the sample was ethnically diverse, Non-Hispanic Whites were overrepresented, which may introduce sampling bias and restrict the generalizability of the findings. Lastly, the exclusion of about 48% of SDH survey respondents from the analytic sample due to missing data may have introduced selection bias in the current analyses.

## 6. Conclusions

Our study highlights discrimination in medical settings among persons with T2D as a pressing health concern. A recent international consensus statement provides key recommendations for addressing diabetes-related stigma and discrimination, emphasizing the need for healthcare institutions to prioritize provider training programs aimed at recognizing and mitigating implicit biases [8]. This initiative advocates for cultivating empathy, person-centered communication, and cultural competence among healthcare providers to create more supportive environments for individuals with T2D. The moderating effect of social support on the link between perceived discrimination and mental HRQoL further underscores the value of integrating social support systems within healthcare settings. Incorporating structured social support services—such as peer support groups, counseling, and mentorship programs—into diabetes care can offer emotional validation and shared understanding. These interventions may bolster resilience against the psychological impact of discrimination, fostering improved mental health outcomes for individuals with T2D.

## Figures and Tables

**Table 1:** Characteristics of study sample (N = 5180).

Characteristic	N (%)
Age (mean, SD)	61.69 (11.27)
Body Mass Index, kg/m^2^ (mean, SD)	34.36 (7.69)
Sex at birth	
Male	2366 (45.68)
Female	2814 (54.32)
Race and ethnicity	
Non-Hispanic White	3804 (73.44)
Non-Hispanic Black	658 (12.70)
Hispanic	459 (8.86)
Other	259 (5.00)
Marital status	
Single	769 (14.85)
Married/living with partner	3000 (57.92)
Widowed/Divorced/Separated	1411 (27.24)
Household income	
<$35,000	1579 (30.48)
$35,000–$74,999	1538 (29.69)
$75,000–$99,999	710 (13.71)
≥$100,000	1411 (27.24)
Education	
High school or less	758 (14.63)
Some college education	1697 (32.76)
College graduate	1376 (26.56)
Advanced degree	1349 (26.04)

**Table 2:** Association between healthcare discrimination and mental HRQoL.

	Model 1a		Model 1b—With Interaction	
	Adjusted Beta (95% CI)	*p*-Value	Adjusted Beta (95% CI)	*p*-Value
Discrimination in healthcare (mEDS)	−0.12 [−0.14, −0.10]	<0.0001	−0.16 [−0.20, −0.12]	<0.0001
Social support (MOS-SSS)	0.03 [0.03, 0.03]	<0.0001	0.02 [0.02, 0.03]	<0.0001
Age	0.08 [0.07, 0.09]	<0.0001	0.08 [0.07, 0.09]	<0.0001
Body Mass Index	−0.03 [−0.04, −0.02]	<0.0001	−0.03 [−0.04, −0.02]	<0.0001
Sex at birth				
Female	[Reference]		[Reference]	
Male	0.09 [−0.08, 0.26]	0.300	0.09 [−0.08, 0.26]	0.308
Race and ethnicity				
Non-Hispanic White	[Reference]		[Reference]	
Non-Hispanic Black	0.29 [0.04, 0.54]	0.022	0.29 [0.04, 0.54]	0.025
Hispanic	0.30 [0.00, 0.59]	0.048	0.30 [0.01, 0.59]	0.046
Other	0.10 [−0.27, 0.47]	0.587	0.11 [−0.26, 0.48]	0.566
Marital status				
Single	[Reference}		[Reference]	
Married/living with partner	−0.27 [−0.53, −0.01]	0.038	−0.26 [−0.52, −0.01]	0.043
Widowed/Divorced/Separated	−0.47 [−0.73, −0.21]	0.0005	−0.47 [−0.73, −0.20]	0.001
Household income				
<$35,000	[Reference]		[Reference]	
$35,000–$74,999	0.89 [0.67, 1.11]	<0.0001	0.88 [0.66, 1.11]	<0.0001
$75,000–$99,999	1.20 [0.92, 1.49]	<0.0001	1.19 [0.90, 1.47]	<0.0001
≥$100,000	1.61 [1.34, 1.87]	<0.0001	1.60 [1.34, 1.87]	<0.0001
Education				
High school or less	[Reference]		[Reference]	
Some college education	0.28 [0.02, 0.53]	0.034	0.28 [0.02, 0.53]	0.034
College graduate	0.52 [0.24, 0.79]	0.0002	0.52 [0.24, 0.79]	0.0002
Advanced degree	0.73 [0.45, 1.02]	<0.0001	0.73 [0.44, 1.02]	<0.0001
mEDS × MOS-SSS (interaction term)	-	-	0.001 [0.0001, 0.0012]	0.0268
Adjusted R^2^	34.7%		31.8%	
Mean Variance Inflation Factor	1.08		1.48	

**Table 3:** Association between healthcare discrimination and physical HRQoL.

	Model 2a		Model 2b—with Interaction	
	Adjusted Beta (95% CI)	*p*-Value	Adjusted Beta (95% CI)	*p*-Value
Discrimination in healthcare (mEDS)	−0.10 [−0.11, −0.08]	<0.0001	−0.10 [−0.14, −0.07]	<0.0001
Social support (MOS-SSS)	0.01 [0.01, 0.01]	<0.0001	0.01 [0.004, 0.016]	0.002
Age	0.03 [0.02, 0.03]	<0.0001	0.03 [0.02, 0.03]	<0.0001
Body Mass Index	−0.07 [−0.08, −0.06]	<0.0001	−0.07 [−0.08, −0.06]	<0.0001
Sex at birth				
Female	[Reference]		[Reference]	
Male	0.27 [0.12, 0.42]	0.0003	0.27 [0.12, 0.42]	0.0003
Race and ethnicity				
Non-Hispanic White	[Reference]		[Reference]	
Non-Hispanic Black	0.03 [−0.18, 0.25]	0.772	0.03 [−0.18, 0.25]	0.776
Hispanic	−0.16 [−0.41, 0.10]	0.229	−0.16 [−0.41, 0.10]	0.230
Other	−0.02 [−0.34, 0.29]	0.890	−0.02 [−0.34, 0.30]	0.894
Marital status				
Single	[Reference}		[Reference]	
Married/living with partner	−0.49 [−0.71, −0.27]	<0.0001	−0.49 [−0.71, −0.27]	<0.0001
Widowed/Divorced/Separated	−0.39 [−0.62, −0.16]	0.0007	−0.39 [−0.62, −0.16]	0.0007
Household income				
<$35,000	[Reference]		[Reference]	
$35,000–$74,999	1.10 [0.91, 1.29]	<0.0001	1.10 [0.91, 1.29]	<0.0001
$75,000–$99,999	1.40 [1.15, 1.64]	<0.0001	1.39 [1.15, 1.64]	<0.0001
≥$100,000	1.68 [1.45, 1.91]	<0.0001	1.68 [1.45, 1.91]	<0.0001
Education				
High school or less	[Reference]		[Reference]	
Some college education	0.42 [0.20, 0.64]	0.0002	0.42 [0.20, 0.64]	0.0002
College graduate	0.86 [0.63, 1.10]	<0.0001	0.86 [0.63, 1.10]	<0.0001
Advanced degree	0.97 [0.73, 1.22]	<0.0001	0.97 [0.73, 1.22]	<0.0001
mEDS X MOS-SSS (interaction term)	-	-	0.00007	0.780
Adjusted R^2^	26.3%		26.3%	
Mean Variance Inflation Factor	1.08		1.48	

**Table 4:** Slope analysis from Haye’s PROCESS 1 model.

Social Support	Effect	SE	T	*p*	95% CI
Low = 40.740 (−1 SD)	−0.136	0.011	−12.075	<0.001	[−0.159, −0.114]
Average = 68.168 (Mean)	−0.119	0.009	−12.728	<0.001	[−0.137, −0.101]
High = 95.597 (+1 SD)	−0.101	0.013	−7.822	<0.001	[−0.127, −0.076]

## Data Availability

The data utilized in this study are accessible to only qualified researchers through the All of Us Research Workbench, via https://workbench.researchallofus.org/login, accessed on 10 July 2024.
